# Management of headache disorders: design of a randomised clinical trial screening for prognostic patient characteristics

**DOI:** 10.1186/1471-2474-8-38

**Published:** 2007-04-26

**Authors:** Willem J De Hertogh, Peter H Vaes, Dirk Devroey, Steven Truijen, William Duquet, Rob Oostendorp

**Affiliations:** 1 Faculty of Physical Education and Physical Therapy, Vrije Universiteit Brussel, Brussels, Belgium; 2 Postgraduate Education in Manual Therapy, Faculty of Medicine and Pharmacy, Vrije Universiteit Brussel, Brussels, Belgium; 3 Department of Health Sciences, University College Antwerp, Antwerp, Belgium; 4 Department of General Practice, University of Brussels, Belgium; 5 Dutch Institute of Allied Health Care, Amersfoort, The Netherlands; 6 Centre for Allied health Sciences, Department of Quality of Care Research, Radboud University Nijmegen Medical Centre, Nijmegen, The Netherlands

## Abstract

**Background:**

Treatment of headache disorders is not always optimal. Patients are treated in multiple ways, and the lack of scientific arguments for referral and the insufficient implementation of guidelines result in unclear treatment strategies.

The coexistence of headache and neck pain can lead to the referral to a musculoskeletal physiotherapist. This treatment can only be successful if an underlying cervical segmental dysfunction is present. In such cases a physical treatment can be a valuable option that should be considered.

The aim of this study is to identify prognostic therapeutic patient characteristics and to increase the number of correct physiotherapy referrals.

**Methods/design:**

This trial is designed to identify patient characteristics which can influence the prognosis of the patient. Patients with recurrent headache and co-existent neck pain are recruited via a multicenter setup. After screening for eligibility, subjects are tested at baseline and randomly allocated to one of two treatment groups. Testing includes the administering of questionnaires (a Headache Diagnosis Questionnaire, Headache Inventory List and the Headache Impact Test (HIT-6)) and physical tests (Thermal Stimuli, Manual Cervical Spine Examination and Pressure Algometry). Treatment groups are a usual care group (UC) administered by the General Practitioner (GP) and a usual care plus musculoskeletal physiotherapy treatment group (UCMT). UC is based on the Dutch GP Guideline for Headache. UCMT consists of the UC plus a combination of exercises and spinal cervical mobilisations. Follow-up measurements consist of the completion of the Headache Inventory List, the HIT-6 and scoring of the global perceived effect (GPE). The latter allowing the distinction between responders (positive effect) and non-responders (no effect or worse). Logistic regression analysis will be used to identify the specific patient characteristics of the responders and the non-responders. The additional value of the musculoskeletal physiotherapy will be examined. Follow-up measurements up to 52 weeks are scheduled.

**Discussion:**

This trial aims to identify prognostic patient characteristics, in order to supply a useful diagnostic tool for all health care workers, dealing with headache sufferers.

## Background

Headache is a common disorder with a high impact, both on the socio-economic level and on the level of the individual sufferer [1-3]. Not all headache patients seek headache treatment, although severe pain and disability have been reported even in large proportions of non-care seekers [4]. Patients do not look for treatment partially out of ignorance of effective treatments or because of previous negative experiences [2,5].

Guidelines for headache management in primary care are available [6,7]. These guidelines provide diagnostic and therapeutic algorithms and stepped treatment plans for the most common headache types. Apparently they are insufficiently applied in daily practice as patients are frequently referred to neurology clinics after no more than one visit to the general practitioners (GP) office [8].

Physical treatments of the cervical spine, provided by musculoskeletal physiotherapists can be considered as a treatment option in headache sufferers. The effects of musculoskeletal physiotherapy for various headache types are reported both in RCT's and systematic reviews [9-16]. The combination of mobilisations and low-load exercises focussing on the cranio-cervical flexion is of particular interest nowadays [9,13]. The conclusions of systematic reviews are not firm due to methodological limitations of the selected studies, but there are indications that musculoskeletal physiotherapy directed to the cervical spine can be beneficial for some headache patients [11,15]. Reductions in headache frequency, intensity and duration of headache attacks have been reported [9,13]. Dowson et al. recommend physical therapy (muscle relaxing and mobilising exercises) for those patients who report neck stiffness [17,18]. The neck-headache relationship is however not always causal: neck pain experienced by migraine patients for example can be a result of sensitization [19]. The co-existence of headache and neck pain and/or the presence of neck stiffness might therefore not be a sufficient indication to prescribe physical treatment of the cervical spine.

These therapeutic uncertainties contribute to patients' treatment dissatisfaction. Relatively high percentages of headache sufferers are not satisfied with the headache care they receive. Harpole and co-workers investigated the headache burden and patient satisfaction with existing management in primary care, using patient-administered surveys. Of the 385 responders, who all contacted a GP because of their headache, 48 % reported problems with headache management and 17 % were dissatisfied with the headache care. Patients with a high headache-related impairment reported more problems with their headache management [20]. Walling et al. found that 26 % were dissatisfied in their sample of 447 migraine patients [21].

The prevalence and burden of headache, the dissatisfaction percentages and the diversity in headache treatments, call for a refinement of headache management.

The identification prior to the start of a treatment, of patients whom might react properly to the treatment, is necessary to reduce the risk of a negative outcome. For this purpose, patient characteristics which predict the treatment outcome (prognostic characteristics) need to be determined.

Prognostic factors are multifactorial. Jull and Stanton analysed the results of 152 CErvicogenic Headache (CEH) patients who participated in one of the three active treatment groups in a headache trial [9]. The prognostic value of socio-demographic factors, headache features, physical impairments of the cervical spine, neck pain and disability and patients' perceptions of influences on their headaches (locus of control) was investigated. They were unable to identify a systematic pattern of prognostic factors. Only the presence of light-headedness appeared to be a clinically relevant predictor of a bad outcome. Of the physical tests only joint pain on palpation had a prognostic value [22]. Of their tests only this palpation could be used for prognostic uses.

Our study has a double aim. Our primary aim is to analyse the prognostic value of a series of physical tests. Our secondary aim is to compare two currently applied treatment strategies, being the usual care (UC) administered by a medical doctor following a stepped guideline [7], and the UC plus a musculoskeletal physiotherapy treatment protocol (UCMT), described by Jull et al [9].

Our research questions are: first, can we identify prognostic patient characteristics, and second, what is the additional value of a musculoskeletal physiotherapy approach in the treatment of headache disorders?

## Methods/design

### Study design

A randomised clinical trial with blinded assessment and unblinded treatment and with a follow-up period of 12 months was developed. The research protocol was approved by the Medical Ethics Committees of the University Hospital of the Vrije Universiteit Brussel (UZ Brussel) and of the University Hospital of Antwerp (UZA).

### Patients selection

Using a multicenter setup, patients will be recruited at GPs offices, from outpatient clinics of the neurology department of the academic hospitals UZ Brussel and UZA and via advertisements.

All participating medical doctors (GP's and neurologists) were contacted and personally informed about the study protocol. Additional information is provided on an informative website [23].

The advertisements contain a weblink to an informative recruitment website [24]. On this site detailed information is provided, followed by an online eligibility screenings procedure.

Different recruitment strategies can result in a heterogenous study population. This will not necessarily affect treatment outcome [25]. Subgroups will be compared at baseline. In case differences are found these will be taken into account in the statistical analysis (see section 'Analyses').

### In- and exclusion criteria

Dutch speaking patients with a combination of recurrent headache and neck pain since minimum two months and at least twice a month with an active help-request (who consider to undergo treatment) are recruited. They have to be at least 18 years old and be willing to participate. Subjects are excluded in case of cluster headache or trigeminal neuralgia. To avoid false positive tests with the quantitative sensory tests (see description of measurements), patients with peripheral neuropathies and co-morbidity of chronic musculoskeletal pain will be excluded. Because of the neck mobility tests, people with rheumatoid arthritis, Down syndrome and/or a history of neck surgery will be excluded. The presence of red flags for headache (warning signs for serious causes of the headache) is an extra exclusion criterion. Pregnant women are also excluded, as pregnancy influences the frequency and intensity of headaches with migraine properties.

To avoid biased patients (with treatment preferences), we excluded patients who received physiotherapy treatment for their headaches during the last 12 months or patients whose prescribed medication was changed during the last two months.

The included headache types are migraine, tension-type headache and CEH. For migraine and tension-type headache the IHS criteria are used [26], for CEH the criteria of the Cervicogenic Headache International Study Group (CHISG) [27].

For the patients recruited via internet sites and advertisements, an additional exclusion criteria is set: a HIT-6 score of at least 56 points. From this level, the HIT-6 scoring advises the patient to contact his/her GP in order to start a treatment [28].

All subjects sign a written informed consent.

### Baseline measurements

Baseline measurements consist of two parts: the completion of questionnaires and physical tests. All baseline measurements are performed by a blinded examiner/rater, as subjects are allocated at random after the baseline measurements (see below: Randomization).

#### A. Questionnaires

All questionnaires can be completed online using PHP-surveyor [29]. In case a participant does not have the opportunity to fill in the questionnaires online, a paper version including an addressed and stamped envelope is provided. The rater is blinded: the online version is completed by the participants themselves, the paper versions are inserted in the computer software by an independent, blinded rater.

##### ■ Headache Diagnosis Questionnaire

This questionnaire was developed to screen for CEH [30]. It consists of 56 questions in total: 34 concerning headache, 15 concerning neck pain and 7 concerning shoulder pain. It registers headache-associated features in a systematic way. Additionally it creates an inventory of pain intensity (Visual Analogue Scale, VAS) of headache, neck pain and shoulder pain (average pain intensity over the last three months and at the moment of fulfilment of the questionnaire), frequency and duration of the headache history. It was developed as a CEH questionnaire and additional questions screening for migraine and tension-type headache characteristics were added. The questionnaire will be validated throughout the trial [30,31].

##### ■ The Headache Impact Test (HIT-6) [32]

The HIT-6 is a short questionnaire, consisting of 6 items: pain intensity, social functioning, role functioning, vitality, cognitive functioning and psychological distress. It has a recall period of four weeks. Scores vary between 36 and 78. Higher scores correspond with a higher headache burden.

The six items are derived from a total pool of 89 items, 54 items from the computer based HIT pool and 35 items that were proposed by headache experts [32]. Its psychometric properties have been investigated extensively [32,33]. Reliability analyses (internal consistency, alternate forms, test-retest) are good to excellent (scores ranging from 0.78 to 0.90). Construct validity has been investigated using the SF-8 as criterion, and negative correlations were found. The HIT-6 is able to differentiate between mild, moderate and severe headache forms. Pain, role functioning and psychological distress are the most differentiating items. A difference of three points is believed to be clinically important (responsiveness). These results were generated in a general population [32], and were recently confirmed in a population of recent headache sufferers recruited in a headache centre [33].

##### ■ Headache Inventory List

This questionnaire is used to register the frequency and intensity of the headache, medication use (both over-the-counter and prescription), headache-related professional care and absenteeism. A similar patient administered questionnaire has been used by Peters et al. [34].

Pain intensity is registered on a VAS for pain at the moment of completion of the questionnaire and for the average pain of the last 4 weeks. Medication intake is monitored, precautionary and treatment medication, both on prescription or over-the-counter.

Headache-related professional care is monitored providing a list of health-care workers who might be contacted by a headache patient (e.g. pharmacist, nurse, and psychologist). Patients have to mark those health-care workers they contacted as well as the frequency of the visits. This registration will allow the analysis of additional therapies that might interfere with the treatment protocol of the study.

Sick leave is scored by the number of absence days.

For all questionnaires a recall period of four weeks is chosen, in correspondence with the recall period of the HIT-6. At baseline and at the last administration of the questionnaires (follow-up at 52 weeks) the absenteeism of the past 12 months is monitored.

#### B. Physical tests

##### Physical examination of the cervical spine

The cervical spine is examined using a manual rotation test and a pain provocation test.

The rotation test is performed for both rotations (left and right) at the C0-2 and C2-7 regions. It is scored for mobility (hyper/hypo or normal), endfeel (too hard, normal, too soft or empty) and for the onset of pain (yes/no). The test is considered positive if two out of three criteria were positive, i.e. hyper- or hypo-mobility, too hard or soft or empty endfeel, and the provocation of pain.

The pain provocation test is the adapted Spurling test (passive lateral flexion, homolateral rotation, extension and axial compression at the end). It is performed on all segments starting at C0-1 and descending till C6-7. It is scored for pain provocation and endfeel. When pain is provoked during this test, a VAS (0 mm – 100 mm) is recorded. If subjects rate their pain > 20 mm on a VAS, this test is considered positive.

These tests can discriminate between asymptomatic controls and patients with neck pain [35]. In this study we apply them to identify an underlying painful segmental dysfunction.

##### Thermal and pressure stimuli

Thermal and pressure stimuli are used to detect sensitisation. Stimuli are applied in cranial and extra-cranial regions. To screen for central sensitization, measurements were also taken on the dorsal side of the index, on the tibialis anterior (pressure) and on the thenar (temperature).

Thermal stimuli will be investigated using the TSA II Thermotest (Medoc Advanced Medical Systems Ltd. 1 Ha'Dekel Street P.O. Box 423, Ramat Yishai 30095 Israel). A thermode of 30 × 30 mm is attached first to the skin of the hand (palmar thenar, C6), next to the mastoid process (C2) and finally to the temple (trigeminal nerve). Starting from a baseline temperature of 32°C, the intensity of the stimulus will vary following the method of limits protocol (+/- 1°C/s). Four modalities are tested: cold and warm sensation (CS and WS) and cold and heat pain detection thresholds (CP and HP). First subjects are asked to press a button as soon a change in temperature is perceived (CS and WS, 4 stimuli). Second they have to push the button when the temperature stimulus is perceived as unpleasant/painful (CP and HP, 3 stimuli). All measurements are performed bilaterally. The average of the repetitions is used for further calculations.

The sequence of the thermal stimuli is chosen to familiarise the subjects with the test: it is reassuring to have the first set of stimuli on the hand and not on the head, which is the site of the complaints. This method has been shown to be reliable [36].

Pressure stimuli are measured using a hand held algometer (Somedic AB, Farsta, Sweden). Pain detection thresholds will be measured on the temple, the mastoid process, the dorsal side of the index and on the tibialis anterior muscle. The average of three measurements will be used for further calculations. Subjects have to report when the feeling of pressure alone changes into a feeling of pressure and pain.

Quantitative Sensory Tests are psychophysical tests. To increase the reliability and ecological validity of the tests, all measurements will be performed at the participants' home.

### Follow-up measurements

After approximately 7, 12, 26 and 52 weeks, participants will be asked to complete the Headache Inventory List and HIT-6 again. For all questionnaires a recall period of four weeks is chosen, in correspondence with the recall period of the HIT-6.

The global perceived effect (GPE) is measured using a 7 point scale, ranging from 'completely recovered' to 'worse than ever'. This way of measuring GPE has been used in similar studies in which the effect of commonly applied therapeutic approaches have been compared [37,38]. Responders (positive effect) and non-responders (no effect or worse) can be identified.

The follow-up measurements are also available online ensuring a blinded rater assessment. The questionnaires which are returned in paper version (from participants without internet connection) will be inserted in the software by an independent blinded rater.

#### Primary and secondary outcome measures

Two primary outcome measures will be used in this study: GPE and the HIT-6 score.

Reduction in headache frequency, headache pain intensity, medication intake, absenteeism and looking for professional help will be used as secondary outcome measures.

### Interventions

The UCMT group will receive a musculoskeletal physiotherapy treatment during 6 weeks following the protocol described by Jull et al. [10,11]. It consists of a combination of spinal mobilisations and exercise therapy. Spinal mobilisations consist of low and/or high-velocity cervical joint mobilization techniques. Each therapist can decide the technique of choice based on his own clinical skills and the patient's situation. Therapeutic exercises consist of low-load endurance exercises, more precisely cranio cervical flexion exercises. A maximum of 12 sessions (twice a week over a period of 6 weeks) is provided. Each session lasts approximately 30 minutes.

All participants receive a letter containing recommendations for treatment, based on the available evidence. This letter is to be handed over to the therapist. After the six weeks treatment period, the therapists will be contacted by phone to check for treatment integrity.

As a guideline for the UC treatment, the protocol from the Dutch College of GPs is used. It consists of a stepped approach to diagnose and treat primary headache disorders. In the Flemish part of Belgium this is the guideline with the lowest access threshold. The GP receives an email containing a weblink to the Dutch College of GPs guideline [7]. In this treatment group treatment integrity will be analysed via the Headache Inventory List, which is to be completed in the follow-up measurements.

### Randomisation

After baseline measurements patients are randomised. Subjects are randomised using blinded envelopes, using a pre-stratification for the headache diagnosis. So, an envelope for migraine, tension-type headache and CEH is provided. Each envelope contains 10 notes, five mentioning usual care and five mentioning usual care plus manual therapy. This to ensure that for each 10 patients with the same diagnosis, the same number of patients is randomised in one of the two treatment groups. Due to the randomisation after testing all tests are performed by a blinded rater.

### Statistical analyses, power and sample size calculation

The prognostic capacity of the initial series of tests (physical examination of the cervical spine and QST) will be calculated by means of a logistic regression analysis, comparing the results of the responders with those of the non-responders. Instead of starting with a full regression model, two known prognostic factors, derived from the study from Jull and Stanton [8] will be included in the analysis a priori. Those two factors are 'light-headedness' and 'joint pain on palpation'. Consequently a backwards stepwise selection will be used, including the two known factors in the smaller models. Differences at baseline between the two treatment groups will be included in the regression models as potential prognostic factors. The plausibility of the signs will be considered to check the logical contribution of each factor in the model. The power of the obtained models will be analysed via the area under the ROC-curve.

Group differences and effect sizes can be calculated for headache intensity and frequency, medication intake, HIT-6 scores and absenteeism. The number of responders and non-responders in both groups will be compared. GPE is a dichotomous variable. Sample size calculations with a significance level of p < 0.05, an event rate of 0.50 in the usual care group and of 0.70 in the usual care plus musculoskeletal physiotherapy group a power of 80% and an equal amount of subjects in both treatment groups result in a sample size of 93 subjects in each treatment group. This results in a total of 186 subjects.

The results of all subjects will be analysed, regardless of their treatment adherence (intention-to-treat analysis).

## Discussion

This study aims to improve the care for headache patients. Therefore we compare two commonly applied therapeutic strategies: the usual care, administered by the GP, and usual care plus musculoskeletal physiotherapy treatment. We primarily want to screen for prognostic patient characteristics.

For the selection of patients we start from the clinical presentation of symptoms: we look for patients with a combination of headache and neck pain. We not only focus on CEH, but allow migraine and tension-type headache patients to be included as well. We believe this is justified because of the common combination of symptoms.

Is it ethical to provide musculoskeletal physiotherapy treatment to patients with migraine symptoms? Several systematic reviews have been performed concerning the value of physical treatments directed to the cervical spine in headache disorders such as migraine [10,11,39]. These reviews were unable to draw firm conclusions due to the methodological quality of the included RCTs. However, there are indications that physical treatments can be beneficial. There is no group that receives only musculoskeletal physiotherapy treatment. The combination with the GP care is allowed at all times. Prohibiting the use of medication, certainly during a headache attack, would be unethical.

We use a diagnostic headache questionnaire. The value of diagnostic questionnaires in comparison with a clinical interview has been studied [40]. Questionnaires have their limitations [40]. They lack the flexibility of a clinical interview, in which questions can be repeated or reformulated whenever a patient does not fully understand their meaning. Then again the phrasing of a questionnaire is more standardised which we consider as preferential. Rasmussen et al. also mention methodological pitfalls. They particularly mention the internal consistency and test-retest repeatability. Reliability scores of our questionnaire are very high (kappa: 0.982, sign.: <0.001), as well as the internal consistency (Cronbach alpha: 0.87) [30]. Considering these methodological aspects we believe that the use of this questionnaire is justified.

We preferred not to include generic questionnaires like the SF-36 in our outcome measures. This was mostly out of practical considerations: the initial set of questionnaires is quite long to complete. Adding another questionnaire can discourage patients to participate. The influence of migraine, tension-type headache and CEH on Quality Of Life (QOL) has been investigated before [41].

Quantitative Sensory Testing using thermal and pressure stimuli have been used to study patho-physiological mechanisms in headache patients [42-46]. General hypersensitivity has been demonstrated to be present in Whiplash patients with moderate to severe symptoms and not in recovered patients or those with mild symptoms [47]. This indicates the potential value of this test in predicting the evolution of a disorder.

The number of participants is the weak point of every clinical study. A pilot running from February 06 to June 06, with only first line recruitments resulted in 15 referred patients. To obtain the required number of patients the way of recruitment was adapted. Second line and advertisement recruitments were also included. The results will be analysed as in any multicenter trial: intercenter differences will be analysed to verify if patients can be pooled.

No training sessions dealing with the study protocol or treatments were organised for the participating medical doctors or therapists. This is a potential weakness of the study protocol. It was impossible to organize these sessions. The referring doctors receive no financial incentives or compensations whatsoever for participation in this study. They participate out of goodwill. Additional time investments would decrease this goodwill. We tried to have their commitment by informing them personally and by providing additional information on a website developed for that purpose [23].

The therapists could not be trained in advance either as it is not known in advance which therapists will participate. This depends on the patient's residence. Therefore the letter with treatment recommendations is provided.

The treatment part of the study protocol is a potential barrier for doctors to participate in the study. Clinicians have treatment preferences. Study protocols require a step back from daily routine, and maybe ask to prescribe a treatment one does not fully support. This can influence the selection of patients: one can refer only those patients who do not fit in the regular schema or where the regular schema was not successful. To anticipate this phenomenon, we contacted all doctors personally, informed them about the study protocol and asked them to collaborate. We labelled one of the treatment groups as the usual care group to link the study protocol with every day practice explicitly.

Patients who are willing to participate are informed prior to the study (written informed consent). Patients with a preference for one of the treatment groups are potentially biased when included.

For these reasons Pfeiffer et al. decided to use a prospective cohort study rather than a randomised controlled trial [48]. In case a study protocol acquires a too low number of patients, using a non-randomised protocol can be an option.

With this study we hope to detect variables that can predict the outcome of the treatment of headache patients, and that consequently can help to improve the care for these patients.

## List of abbreviations

HIT-6: Headache Impact Tests, 6 items

UC: Usual Care

UCMT: Usual Care plus Musculoskeletal Physiotherapy Treatment

GP: General Practitioner

RCT: Randomised Clinical Trial

CEH: CErvicogenic Headache

IHS: International Headache Society

CHISG: Cervicogenic Headache International Study Group

VAS: Visual Analogue Scale

QST: Quantitative Sensory Testing.

CS: Cold Sensation

WS: Warm Sensation

CP: Cold Pain

HP: Heat Pain

GPE: Global Perceived Effect

QOL: Quality Of Life

SF-8: Short form-8 quality of life questionnaire

SF-36: Short form-36 quality of life questionnaire

## Competing interests

The author(s) declare that they have no competing interests.

## Authors' contributions

WDH, PV, RO, DD and WD participated in the design and coordination of the study. WD and ST participate in the statistical calculations of the results.

All authors read and approved the final manuscript.

## Pre-publication history

The pre-publication history for this paper can be accessed here:


